# Anxiety in oncology outpatients is associated with perturbations in pathways identified in anxiety focused network pharmacology research

**DOI:** 10.1007/s00520-023-08196-2

**Published:** 2023-11-28

**Authors:** Kate Oppegaard, Kord M. Kober, Carolyn Harris, Joosun Shin, Lisa Morse, Alejandra Calvo-Schimmel, Steven M. Paul, Bruce A. Cooper, Yvette P. Conley, Marilyn Hammer, Vasuda Dokiparthi, Jon D. Levine, Christine Miaskowski

**Affiliations:** 1 Department of Physiological Nursing, School of Nursing, University of California, 2 Koret Way - N631Y, San Francisco, CA USA; 2 School of Nursing, University of Pittsburgh, Pittsburgh, PA USA; 3 Dana-Farber Cancer Institute, Boston, MA USA; 4 School of Medicine, University of California, San Francisco, CA USA

**Keywords:** Anxiety, Cancer, Chemotherapy, Pathway impact analysis

## Abstract

**Purpose:**

Evaluate for perturbed signaling pathways associated with subgroups of patients with low versus high levels of state anxiety. These pathways were compared to the pathways identified across eight network pharmacology studies of the anxiolytic effect(s) of a variety of compounds.

**Methods:**

Adult outpatients had a diagnosis of breast, gastrointestinal, gynecological, or lung cancer; had received chemotherapy within the preceding four weeks; and were scheduled to receive at least two additional cycles of chemotherapy. Latent profile analysis was used to identify subgroups of patients with distinct anxiety profiles based on Spielberger State Anxiety Inventory scores that were obtained six times over two cycles of chemotherapy. Blood samples were processed using RNA sequencing (i.e., RNA-seq sample, *n* = 244) and microarray (i.e., microarray sample; *n* = 256) technologies. Pathway perturbations were assessed using pathway impact analysis. Fisher’s combined probability method was used to combine test results using a false discovery rate of 0.01.

**Results:**

In the RNA-seq sample, 62.3% and 37.7% of the patients were in the low- and high-anxiety classes, respectively. In the microarray sample, 61.3% and 38.7% were in the low and high-anxiety classes, respectively. Forty-one perturbed signaling pathways were identified. Eight of these pathways were common to those identified in the network pharmacology studies.

**Conclusions:**

Findings increase our knowledge of the molecular mechanisms that underlie anxiety in patients receiving chemotherapy. This study provides initial insights into how anxiety in patients with cancer may share common mechanisms with anxiety in patients with other clinical conditions.

**Supplementary Information:**

The online version contains supplementary material available at 10.1007/s00520-023-08196-2.

## Introduction

Between 16 and 42% of patients report high levels of anxiety at the time of their cancer diagnosis and during its treatment [1–4]. In patients receiving chemotherapy, high levels of anxiety are associated with treatment delays [5], prolonged duration of co-occurring symptoms [6], and decrements in quality of life [7]. In addition, untreated anxiety may contribute to disease recurrence and decreased survival [8]. While prevalence rates for and impact of anxiety were the subject of several reviews [1–4], less is known about the underlying mechanisms for this symptom in patients receiving chemotherapy. This knowledge is required for the development of targeted and effective interventions.

While the neurobiological mechanisms that contribute to anxiety are not fully understood, the extended amygdala appears to be an area from which brain loci form circuits to mediate and modulate anxiety-like behavior [9]. Other areas of the brain involved in anxiety include the medial prefrontal cortex, hippocampus, locus coeruleus, raphe nucleus, and the ventral tegmental area [9]. Communication among these circuits is facilitated through well-characterized neurotransmitters and neuromodulators [9, 10]. Anxiety symptoms may occur as a result of changes or deficiencies in processing in these circuits, specifically, imbalances in neurotransmitter regulation and perturbations in downstream signaling pathways [9].

Pathway analysis is one method that can be used to evaluate the molecular mechanisms that underlie symptoms [11]. While pathway analysis was used to evaluate common symptoms associated with cancer and its treatments (e.g., fatigue [12], cancer-related cognitive impairment [13], nausea [14]), this approach has not been used to evaluate anxiety in patients with or without cancer. However, pathway analysis was used as a component of network pharmacology studies of anxiety and associated therapeutics [15–22]. In brief, network pharmacology is a bioinformatics approach that aims to identify the molecules, targets, and signaling pathways that different compounds (e.g., Chinese medicines, herbal preparations) use to exert their effect(s) [23, 24]. Using database inquiries, overlapping genes known to be associated with a given phenotype and genes derived from the bioactive components of a compound of interest are identified [21]. Then, pathway analysis is used to elucidate the biological pathways that contribute to the therapeutic effects of the compound(s) of interest. Network pharmacology offers an opportunity to gain knowledge about biological mechanisms that underlie symptoms or diseases that can serve as targets for interventions [25].

Eight studies were identified that used a network pharmacology approach to evaluate the mechanism(s) associated with the anxiolytic effect(s) of a variety of compounds [15–22]. Across these eight studies, human genes associated with various anxiety phenotypes were identified using several database and the following key words: “anxiety,” “generalized anxiety disorder,” or “anxiety disorder(s)” [15–22]. For example, in one study [18], the searches included breast cancer–related anxiety and the anxiolytic mechanism(s) of Baihedihuang decoction (i.e., a combination of Chinese medicines) [18]. Seventeen signaling pathways appear to be involved in the mechanisms that underlie the anxiolytic effects of Baihedihuang decoction in patients with breast cancer (Table 1). Across the other seven studies, similar analyses were done for Jujebee seed [15], Rehmannia root and Chinese arborvitae kernel [19], Roman chamomile [16], Jiu Wei Zhen Xin formula [20], rose-bergamot essential oil [22], Wendan decoction [17], and Bupleurum [21]. Across these eight studies, the neuroactive ligand-receptor interaction and the serotonergic synapse pathways were identified as common pathways. Based on these findings, it is reasonable to hypothesize that these pathways may be involved in clinical levels of anxiety. Additional pathways identified in one or more of these studies are summarized in Table 1 [15–22].

**Table 1:** Summary of pathways identified in network pharmacology studies focused on the identification of the mechanism(s) associated with the anxiolytic effect(s) of a variety of compounds

	First author, year, and phenotypic search query							
Pathway name	Chen 2020^1^ “Anxiety”	Liu 2021^2^ “Anxiety disorders”	Jia 2021^3^ “Anxiety disorder”	Li 2020^4^ “Breast cancer” and “anxiety”	Shao 2022^5^ “Generalized anxiety disorder”	Xin 2022^6^ “Anxiety disorder”	Jin 2022^7^ “Generalized anxiety disorder”	Wu 2022^8^ “Anxiety”
Neuroactive ligand-receptor interaction	x	x	x	x	x	x	x	x
Serotonergic synapse	x	x	x	x	x	x	x	x
cAMP signaling		x	x	x	x	x	x	x
Calcium signaling		x	x		x	x	x	x
Retrograde endocannabinoid signaling	x	x	x		x	x		
Morphine addiction	x		x	x	x	x		x
Nicotine addiction	x		x		x	x		x
Prostate cancer	x		x	x		x		
Gap junction	x	x		x		x		
Estrogen signaling				x	x			x
Dopaminergic synapse			x		x	x		x
GABAergic synapse	x				x	x		x
cGMP-PKG signaling		x	x	x				x
Cocaine addiction			x		x	x		
Amphetamine addition			x		x			x
Alcoholism			x	x		x		x
AGE-RAGE signaling pathway in diabetic conditions	x		x					
Pathways in cancer		x		x		x		
Salivary secretion		x						x
PI3K-Akt signaling		x					x	
Taste transduction	x							x
Adrenergic signaling in cardiomyocytes		x						x
MAPK signaling		x					x	
Renin secretion			x					x
Prolactin signaling			x				x	
Regulation of lipolysis in adipocytes			x					x
Rap1 signaling						x	x	
Phospholipase D signaling						x	x	
Proteoglycans in cancer		x		x				
Non-alcoholic fatty liver disease	x							
Longevity regulating	x							
TNF-signaling	x							
Non-small cell lung cancer		x						
Glioma		x						
Small cell lung cancer		x						
Hepatitis C		x						
MicroRNAs in cancer		x						
Oxytocin signaling		x						
Insulin resistance			x					
Alzheimer disease			x					
Sphingolipid signaling			x					
Pathways of neurodegeneration — multiple diseases						x		
Chemical carcinogenesis — receptor activation						x		
EGRF tyrosine kinase inhibitor resistance						x		
Drug metabolism — cytochrome P450						x		
Arginine and proline metabolism						x		
HIF-1 signaling							x	
Thyroid hormone signaling							x	
Relaxin signaling							x	
Ras signaling							x	
FoxO signaling							x	
Chemokine signaling							x	
Cholinergic synapse				x				x
Vascular smooth muscle contraction				x				x
Gastric acid secretion								x
VEGF signaling				x				
Synaptic vesicle cycle				x				
Oocyte meiosis				x				
Chemical carcinogenesis — DNA adducts				x				

Pathways listed were reported in the main article. In some cases, study authors note that only top pathways were reported, and/or additional pathways were reported in supplemental files

In our previous latent profile analysis (LPA) [26], four subgroups of patients with distinct anxiety profiles were identified, namely, low, moderate, high, and very high. Using an extreme phenotype approach, the purpose of this study was to evaluate for perturbed signaling pathways associated with low versus high anxiety classes (i.e., high and very high combined). Then, these pathways were compared to the pathways identified across the eight network pharmacology studies of the anxiolytic effect(s) of a variety of compounds [15–22]. We hypothesized that common pathways would be found across the current and previous studies [15–22].

## Methods

### Patients and settings

This analysis is part of a larger, longitudinal study of the symptom experience of oncology outpatients receiving chemotherapy. Eligible patients were ≥ 18 years of age; had a diagnosis of breast, gastrointestinal, gynecological, or lung cancer; had received chemotherapy within the preceding four weeks; were scheduled to receive at least two additional cycles of chemotherapy; were able to read, write, and understand English; and gave written informed consent. Patients were recruited from two comprehensive cancer centers, one Veteran’s Affairs hospital, and four community-based oncology programs.

### Study procedures

The study was approved by the Committee on Human Research at the University of California, San Francisco, and the Institutional Review Board at each of the study sites. Of the 2234 patients approached, 1343 consented to participate (60.1% response rate). The major reason for refusal was being overwhelmed with their cancer treatment. Eligible patients were approached in the infusion unit during their first or second cycle of chemotherapy by a member of the research team to discuss study participation and obtain written informed consent.

Patients completed the state anxiety measure a total of six times over two cycles of chemotherapy (i.e., prior to chemotherapy administration, approximately 1 week after chemotherapy administration, approximately 2 weeks after chemotherapy administration). All of the other measures and collection of blood for ribonucleic acid (RNA) isolation were done at enrollment (i.e., prior to the second or third cycle of chemotherapy). For this study, a total of 717 patients provided a blood sample for the analyses. Of these 717 patients, 357 had their samples processed using RNA sequencing (i.e., RNA-seq sample), and 360 had their samples processed using microarray (i.e., microarray sample) technologies.

### Instruments

#### Demographic and clinical characteristics

Patients completed a demographic questionnaire, Karnofsky Performance Status (KPS) scale [27], Self-Administered Comorbidity Questionnaire (SCQ) [28], and Alcohol Use Disorders Identification test (AUDIT) [29]. The toxicity of each patient’s chemotherapy regimen was rated using the MAX2 index [30]. Medical records were reviewed for disease and treatment information.

#### Anxiety measure

The 20-items on the Spielberger State Anxiety Inventory (STAI-S) were rated from 1 to 4 [31]. The STAI-S measures a person’s temporary anxiety response to a specific situation or how anxious or tense a person is “right now” in a specific situation. A cut-off score of ≥ 32.2 indicates a high level of state anxiety. Its Cronbach’s alpha was 0.96.

### Data analysis

#### Creation of the anxiety classes

As reported previously [26], LPA was used to identify unobserved latent classes with distinct anxiety profiles over the six assessments, using the patients’ state anxiety scores. Four latent classes were identified and named low, moderate, high, and very high. For the current analysis, using an extreme phenotype approach, patients in the moderate group were excluded, and the remaining patients were classified into two state anxiety groups (i.e., low and high [combined high and very high classes]; Supplemental Figure 1).

#### Imputation process

Missing data for demographic and clinical characteristics were imputed by the k-nearest-neighbors method, with *k* = 9. For continuous variables, the Euclidean distance was used to find the nearest neighbors. The imputed value was the weighted average of the nearest neighbors, with each weight originally exp(-dist(x,j)), after which the weights were scaled to one. For categorical variables, distance was 0 if the target and the neighbor had the same value and 1 if they did not. The imputed value was the mode of the nearest neighbors.

#### Demographic and clinical data

Demographic and clinical data from the two patient samples (i.e., RNA-seq, microarray) were analyzed separately. Differences in demographic and clinical characteristics between the patients in the low and high classes were evaluated using parametric and non-parametric tests. Significance was assessed at a *p*-value of < .05. In order to not overfit the regression models, the number of demographic and clinical characteristics selected for inclusion was based on the sample size for the smaller of the two latent classes. Characteristics included in the final model were selected using a backwards stepwise logistic regression approach based on the likelihood ratio test. The area under the curve of the receiver operating characteristic curves was used to gauge the overall adequacy of the logistic regression model for each sample [32]. All of these analyses were performed using R version 4.0.5 [33].

#### Differential expression and PIA

Details on the gene expression methods and pathway impact analyses (PIA) are described elsewhere [12]. In brief, differential expression was quantified using empirical Bayes models that were implemented separately for each sample (i.e., using edgeR [34] for the RNA-seq sample and limma [35] for the microarray sample). These analyses were adjusted for select demographic and clinical characteristics, as well as surrogate variables (i.e., variations due to unmeasured sources) [36]. Expression loci were annotated with Entrez gene identifiers. Gene symbols were derived from the HUGO Gene Nomenclature Committee resource database [37]. The differential expression results were summarized as the log fold-change and *p*-value for each gene. Only genes that had a common direction of expression (i.e., the same sign for the log fold-change) across the two samples were retained for subsequent analyses. Common genes were matched using gene symbol.

The PIA included the results of the differential expression analyses for all of the genes (i.e., cutoff free) that had a common direction of differential expression to determine probability of pathway perturbations (pPERT) using Pathway Express (version 2.18.0) [38]. A total of 225 signaling pathways were defined using the Kyoto Encyclopedia of Genes and Genomes (KEGG) database [39]. For each sample, a separate test was performed for each pathway. Next, Fisher’s combined probability method was used to combine these test results to obtain a single test (global) of the null hypothesis [40]. The significance of the combined transcriptome-wide PIA was assessed using a false discovery rate (FDR) of 0.01 under the Benjamini-Hochberg procedure [41]. Results were compared to pathways identified in previous network pharmacology research studies focused on the identification of mechanism(s) associated with the anxiolytic effect(s) of a variety of compounds [15–22]. Then, these perturbed pathways were grouped and evaluated using the KEGG database categories.

## Results

### RNA-seq performance

After excluding patients in the moderate class and applying quality controls, 244 patients in the RNA-seq sample had data available for analysis (Supplemental Figure 2). Of these, 62.3% were in the low class and 37.7% were in the high class. Median library threshold size was 9,274,838 reads and 17,714 genes were included in the final analysis. The common dispersion was estimated as 0.214 yielding a biological coefficient of variation of 0.462 [42].

### Microarray performance

After excluding patients in the moderate class and applying quality controls, 256 patients in the microarray sample had data available for analysis (Supplemental Figure 2). Of these, 61.3% were in the low class and 38.7% were in the high class. All of these samples demonstrated good hybridization performance for biotin, background negative, and positive control assays on the arrays. Following quality control filters, 43,900 loci were included in the final analysis.

### Demographic and clinical characteristics

Of the 244 patients in the RNA-seq sample (Table 2), compared to the low class, the high class was younger; more likely to be female; more likely to live alone; and had a lower annual income. In addition, the high class had a lower performance status; a higher number of comorbidities; a higher comorbidity burden; a higher MAX2 score; were less likely to have a diagnosis of gastrointestinal cancer; were more likely to have a diagnosis of lung cancer; were more likely to have a longer chemotherapy cycle length; and were more likely to self-report diagnoses of lung disease, depression, or back pain.

**Table 2:** Differences in demographic and clinical characteristics at enrollment between the low anxiety and high anxiety classes in the RNA-seq sample

Characteristic	Low Anxiety (1) 62.3% (*n* = 152)	High Anxiety (2) 37.7% (*n* = 92)	Statistics
	Mean (SD)	Mean (SD)	
Age (years)	58.6 (11.8)	53.2 (12.6)	*t* = 3.34, *p* <0.001
Education (years)	16.4 (3.1)	15.8 (3.1)	*t* = 1.56, *p* = 0.119
Body mass index (kg/m^2^)	25.9 (4.6)	26.6 (6.2)	*t* = − 0.94, *p* = 0.351
KPS score	81.6 (12.1)	72.9 (11.3)	*t* = 5.59, *p* < 0.001
Number of comorbidities	2.2 (1.4)	3.0 (1.7)	*t* = − 3.83, *p* < 0.001
SCQ score	5.0 (3.0)	7.0 (4.1)	*t* = − 4.25, *p* < 0.001
AUDIT score	2.8 (1.8)	2.8 (2.4)	*t* = − 0.11, *p* = 0.913
Time since diagnosis (years)	1.6 (2.9)	1.2 (1.8)	*U*, *p* = 0.811
Time since diagnosis (years, median)	0.4	0.5	
Number of prior cancer treatments	1.5 (1.4)	1.5 (1.3)	*t* = 0.10, *p* = 0.921
Number of metastatic sites including lymph node involvement	1.1 (1.1)	1.1 (1.3)	*t* = − 0.09, *p* = 0.928
Number of metastatic sites excluding lymph node involvement	0.7 (1.0)	0.7 (1.1)	*t* = − 0.35, *p* = 0.729
MAX2 score	0.17 (0.08)	0.19 (0.08)	*t* = − 2.37, *p* = 0.019
Hemoglobin (g/dL)	11.5 (1.4)	11.3 (1.4)	*t* = 1.03, *p* = 0.303
Hematocrit (%)	34.4 (3.9)	33.7 (4.1)	*t* = 1.32, *p* = 0.188
	% (*n*)	% (*n*)	
Gender			FE, *p* = 0.003
Female	74.3 (113)	90.2 (83)	
Male	25.7 (39)	9.8 (9)	
Ethnicity			*X*^2^ = 5.97, *p* =
Asian or Pacific Islander	17.1 (26)	12.0 (11)	0.113
Black	7.9 (12)	6.5 (6)	
Hispanic, Mixed, or Other	9.2 (14)	19.6 (18)	
White	65.8 (100)	62.0 (57)	
Married or partnered (% yes)	65.8 (100)	55.4 (51)	FE, *p* = 0.134
Lives alone (% yes)	18.4 (28)	34.8 (32)	FE, *p* = 0.006
Childcare responsibilities (% yes)	21.7 (33)	26.1 (24)	FE, *p* = 0.440
Care of adult responsibilities (% yes)	7.9 (12)	6.5 (6)	FE, *p* = 0.804
Currently employed (% yes)	34.9 (53)	28.3 (26)	FE, *p* = 0.324
Income			*U*, *p* = 0.007
< $30,000	13.8 (21)	31.5 (29)	1 > 2
$30,000 to < $70,000	19.7 (30)	21.7 (20)	
$70,000 to < $100,000	27.0 (41)	14.1 (13)	
≥ $100,000	39.5 (60)	32.6 (30)	
Specific comorbidities (% yes)			
Heart disease	5.3 (8)	3.3 (3)	FE, *p* = 0.542
High blood pressure	32.9 (50)	33.7 (31)	FE, *p* = 1.000
Lung disease	5.3 (8)	16.3 (15)	FE, *p* = 0.006
Diabetes	9.9 (15)	13.0 (12)	FE, *p* = 0.528
Ulcer or stomach disease	3.9 (6)	4.3 (4)	FE, *p* = 1.000
Kidney disease	0.7 (1)	2.2 (2)	FE, *p* = 0.559
Liver disease	5.2 (8)	6.5 (6)	FE, *p* = 0.778
Anemia or blood disease	7.9 (12)	15.2 (14)	FE, *p* = 0.088
Depression	9.2 (14)	43.5 (40)	FE, *p* <0.001
Osteoarthritis	11.8 (18)	12.0 (11)	FE, *p* = 1.000
Back pain	21.7 (33)	45.7 (42)	FE, *p* <0.001
Rheumatoid arthritis	5.9 (9)	5.3 (5)	FE, *p* = 1.000
Exercise on a regular basis (% yes)	69.1 (105)	58.7 (54)	FE, *p* = 0.127
Smoking current or history of (% yes)	29.6 (45)	42.4 (39)	FE, *p* = 0.052
Cancer diagnosis			*X*^2^ = 16.19, *p* = 0.001
Breast	40.1 (61)	40.2 (37)	NS
Gastrointestinal	42.1 (64)	22.8 (21)	1 > 2
Gynecological	13.2 (20)	21.7 (20)	NS
Lung	4.6 (7)	15.2 (14)	1 < 2
Type of prior cancer treatment			*X*^2^ = 2.87, *p*= 0.413
No prior treatment	28.3 (43)	23.9 (22)	
Only surgery, CTX, or RT	41.4 (63)	51.1 (47)	
Surgery & CTX, or surgery & RT, or CTX & RT	19.1 (29)	13.0 (12)	
Surgery & CTX & RT	11.2 (17)	12.0 (11)	
CTX cycle length			*U*, *p* <0.001 1 < 2
14-day cycle	57.9 (88)	34.8 (32)	
21-day cycle	35.5 (54)	56.5 (52)	
28-day cycle	6.6 (10)	8.7 (8)	
Emetogenicity of CTX			*U*, *p* = 0.486
Minimal/low	14.5 (22)	18.5 (17)	
Moderate	69.7 (106)	56.5 (52)	
High	15.8 (24)	25.0 (23)	
Antiemetic regimens			*X*^2^ = 6.10, *p* = 0.107
None	5.9 (9)	4.3 (4)	
Steroid alone or serotonin receptor antagonist alone	18.4 (28)	18.5 (17)	
Serotonin receptor antagonist and steroid	54.6 (83)	42.4 (39)	
NK-1 receptor antagonist and two other antiemetics	21.1 (32)	34.8 (32)	

*AUDIT* Alcohol Use Disorders Identification Test, *CTX* chemotherapy, *FE* Fisher’s exact test, g/dL grams per deciliter, *kg* kilograms, *KPS* Karnofsky Performance Status, *m*^*2*^ meter squared, *NK-1* neurokinin-1, *NS* not significant, *RNA-seq* ribonucleic acid sequencing, *RT* radiation therapy, *SCQ* Self-administered Comorbidity Questionnaire, *SD* standard deviation, *U* Mann-Whitney *U* test

Of the 256 patients in the microarray sample (Table 3), compared to the low class, the high class was younger; were more likely to report Black, or Hispanic, mixed or other ethnicity; were less likely to report White ethnicity; were less likely to be married or partnered; were less likely to be employed; had a lower annual income; and had fewer years of education. In addition, the high class had a higher body mass index; a lower performance status; a higher number of comorbidities; a higher comorbidity burden; were more likely to self-report a diagnosis of depression or back pain; were more likely to have had surgery, chemotherapy, and radiation therapy; and were more likely to receive an antiemetic regimen that included a neurokinin-1 receptor antagonist and two other antiemetics.

**Table 3:** Differences in demographic and clinical characteristics at enrollment between the low anxiety and high anxiety classes in the microarray sample

Characteristic	Low anxiety (1) 61.3% (*n* = 157)	High anxiety (2) 38.7% (*n* = 99)	Statistics
	Mean (SD)	Mean (SD)	
Age (years)	58.6 (10.8)	55.3 (12.4)	*t* = 2.26, *p* = 0.025
Education (years)	16.8 (2.8)	15.6 (2.9)	*t* = 2.92, *p* = 0.004
Body mass index (kg/m^2^)	25.8 (5.5)	27.6 (6.9)	*t* = − 2.20, *p* = 0.029
KPS score	82.7 (10.8)	76.2 (11.0)	*t* = 4.66, *p* <0.001
Number of comorbidities	2.2 (1.2)	2.9 (1.5)	*t* = − 3.89, *p* <0.001
SCQ score	4.9 (2.5)	6.6 (3.2)	*t* = − 4.56, *p* <0.001
AUDIT score	2.9 (1.8)	3.0 (2.8)	*t* = − 0.31, *p* = 0.756
Time since diagnosis (years)	2.1 (3.7)	2.2 (3.5)	U, *p* = 0.527
Time since diagnosis (median)	0.4	0.5	
Number of prior cancer treatments	1.7 (1.6)	2.1 (1.7)	*t* = − 1.90, *p* = 0.058
Number of metastatic sites including lymph node involvement	1.3 (1.3)	1.3 (1.3)	*t* = 0.06, *p* = 0.951
Number of metastatic sites excluding lymph node involvement	0.8 (1.1)	0.8 (1.2)	*t* = 0.58, *p* = 0.566
MAX2 score	0.17 (0.08)	0.17 (0.09)	*t* = − 0.36, *p* = 0.716
Hemoglobin (g/dL)	11.7 (1.4)	11.8 (1.5)	*t* = − 0.59, *p* = 0.557
Hematocrit (%)	34.8 (4.1)	35.1 (4.1)	*t* = − 0.56, *p* = 0.576
	% (*n*)	% (*n*)	
Gender			FE, *p* = 0.205
Female	76.4 (120)	83.8 (83)	
Male	23.6 (37)	16.9 (16)	
Ethnicity			X^2^ = 21.61, *p* < 0.001
Asian or Pacific Islander	12.1 (19)	9.1 (9)	NS
Black	5.1 (8)	13.1 (13)	1 < 2
Hispanic, Mixed, or Other	4.5 (7)	19.2 (19)	1 < 2
White	78.3 (123)	58.5 (58)	1 > 2
Married or partnered (% yes)	75.2 (118)	46.5 (46)	FE, *p* <0.001
Lives alone (% yes)	17.8 (28)	26.3 (26)	FE, *p* = 0.118
Childcare responsibilities (% yes)	21.7 (34)	27.3 (27)	FE, *p* = 0.366
Care of adult responsibilities (% yes)	5.7 (9)	10.1 (10)	FE, *p* = 0.225
Currently employed (% yes)	43.9 (69)	17.2 (17)	FE, *p* <0.001
Income			U, *p* <0.001 1 > 2
< $30,000	10.8 (17)	41.4 (41)	
$30,000 to < $70,000	17.8 (28)	19.2 (19)	
$70,000 to < $100,000	22.3 (35)	15.2 (15)	
≥ $100,000	49.0 (77)	24.2 (24)	
Specific comorbidities (% yes)			
Heart disease	5.7 (9)	4.0 (4)	FE, *p* = 0.771
High blood pressure	25.5 (40)	36.4 (36)	FE, *p* = 0.069
Lung disease	11.5 (18)	9.1 (9)	FE, *p* = 0.677
Diabetes	8.9 (14)	9.1 (9)	FE, *p* = 1.000
Ulcer or stomach disease	4.5 (7)	6.1 (6)	FE, *p* = 0.572
Kidney disease	0.6 (1)	1.0 (1)	FE, *p* = 1.000
Liver disease	7.0 (11)	6.1 (6)	FE, *p* = 1.000
Anemia or blood disease	11.5 (18)	18.2 (18)	FE, *p* = 0.143
Depression	8.9 (14)	50.5 (50)	FE, *p* <0.001
Osteoarthritis	10.8 (17)	13.1 (13)	FE, *p* = 0.690
Back pain	21.0 (33)	33.3 (33)	FE, *p* = 0.039
Rheumatoid arthritis	3.2 (5)	5.1 (5)	FE, *p* = 0.516
Exercise on a regular basis (% yes)	77.0 (121)	66.7 (66)	FE, *p* = 0.083
Smoking current or history of (% yes)	37.6 (59)	40.4 (40)	FE, *p* = 0.693
Cancer diagnosis			*X*^2^ = 4.53, *p* =
Breast	35.7 (56)	46.5 (46)	0.210
Gastrointestinal	26.8 (42)	27.3 (27)	
Gynecological	21.7 (34)	17.2 (17)	
Lung	15.9 (25)	9.1 (9)	
Type of prior cancer treatment			*X*^2^ = 8.22, *p* =
No prior treatment	24.2 (38)	14.1 (14)	0.042
Only surgery, CTX, or RT	39.5 (62)	43.4 (43)	NS
Surgery & CTX, or surgery & RT, or CTX & RT	22.3 (35)	17.2 (17)	NS
Surgery & CTX & RT	14.0 (22)	25.3 (25)	NS 1 < 2
CTX cycle length			*U*, *p* = 0.818
14-day cycle	36.3 (57)	36.4 (36)	
21-day cycle	56.1 (88)	53.5 (53)	
28-day cycle	7.6 (12)	10.1 (10)	
Emetogenicity of CTX			*U*, *p* = 0.491
Minimal/low	22.9 (36)	27.3 (27)	
Moderate	58.6 (92)	55.6 (55)	
High	18.5 (29)	17.2 (17)	
Antiemetic regimens			*X*^2^ = 11.05, *p* = 0.011
None	12.7 (20)	12.1 (12)	NS
Steroid alone or serotonin receptor antagonist alone	25.5 (40)	17.2 (17)	NS
Serotonin receptor antagonist and steroid	45.9 (72)	37.4 (37)	NS
NK-1 receptor antagonist and two other antiemetics	15.9 (25)	33.3 (33)	1 < 2

*AUDIT* Alcohol Use Disorders Identification Test, *CTX* chemotherapy, *FE* Fisher’s exact test, *g/dL* grams per deciliter, *kg* kilograms, *KPS* Karnofsky Performance Status, *m*^*2*^ meter squared, *NK-1* neurokinin-1, *NS* not significant, *RT* radiation therapy, *SCQ* Self-administered Comorbidity Questionnaire, *SD* standard deviation, *U* Mann-Whitney *U* test

### Logistic regression analyses

In the logistic regression analysis for the RNA-seq sample (Table 4), eight variables were included in the initial model, and seven of them were retained in the final model (i.e., age, male gender, lives alone, KPS score, MAX2 score, self-reported diagnosis of depression, self-reported diagnosis of back pain) and used as covariates in the gene expression analysis. In the logistic regression analysis for the microarray sample, nine variables were included in the initial model, and six of them were retained in the final model (i.e., age, married or partnered, employed, body mass index, KPS score, self-reported diagnosis of depression) and used as covariates in the gene expression analysis.

**Table 4:** Multiple logistic regression analyses predicting membership in the high anxiety class

RNA-seq sample (*n* = 224)			
Predictors	Odds ratio	95% CI	*p* value
Age	0.96	0.94, 0.99	0.004
Male gender	0.40	0.15, 0.99	0.058
Lives alone	2.42	1.15, 5.17	0.021
Karnofsky Performance Status score	0.96	0.94, 0.99	0.008
MAX2 score	49.87	0.86, 3293.25	0.062
Self-reported diagnosis of depression	5.99	2.79, 13.52	< 0.001
Self-reported diagnosis of back pain	2.48	1.25, 4.94	0.000
Overall model fit: AUC of the ROC = 0.823			
Microarray sample (*n* = 256)			
Predictors	Odds ratio	95% CI	*p* value
Age	0.98	0.95, 1.00	0.087
Married or partnered	0.30	0.15, 0.58	0.001
Employed	0.32	0.15, 0.66	0.003
Body mass index	1.06	1.00, 1.11	0.040
Karnofsky Performance Status score	0.97	0.94, 0.99	0.018
Self-reported diagnosis of depression	8.70	4.22, 18.95	< 0.001
Overall model fit: AUC of the ROC = 0.845			

*AUC* area under curve, *CI* confidence interval, *RNA-seq* ribonucleic acid-sequencing, *ROC* receiver operating characteristic

### Perturbed pathways

For the RNA-seq sample, two surrogate variables were identified and included in the final differential expression model. For the microarray sample, zero surrogate variables were identified. For both samples, a total of 4344 genes were included in the PIA analyses. Using Fisher’s combined probability method, across the two samples, 41 KEGG signaling pathways were significantly perturbed at an FDR of < 0.01 (Supplemental Table 1).

These 41 pathways were compared to the pathways identified in network pharmacology studies focused on elucidation of the mechanism(s) associated with the anxiolytic effect(s) of a variety of compounds (Table 1) [15–22]. In total, eight of the perturbed pathways were common to the pathways identified in the pharmacology studies (Table 5). These eight pathways were grouped into their six respective KEGG database categories, namely, signaling molecules and interaction, nervous system, cancer: overview, signal transduction, neurodegenerative disease, and circulatory system. Specifically, these common pathways included neuroactive ligand-receptor interaction, serotonergic synapse, pathways in cancer, phosphoinositide-3-kinase/Akt (PI3K/Akt) signaling, mitogen-activated protein kinase (MAPK) signaling, pathways of neurodegeneration - multiple diseases, Alzheimer disease (AD), and vascular smooth muscle contraction. Using the KEGG database categories for the common pathways, in the current study, five additional perturbed pathways were identified, namely, cytokine-cytokine receptor interaction, transcriptional misregulation in cancer, prion disease, Huntington disease, and cardiac muscle contraction (Table 5).

**Table 5.** Perturbed pathways for the low anxiety versus high anxiety classes that were common to and distinct from the pathways identified in network pharmacology studies focused on the identification of the mechanism(s) associated with the anxiolytic effect(s) of a variety of compounds

Common pathways			Distinct pathways		
Pathway ID	KEGG pathway name	Combined analysis statistics	Pathway ID	KEGG pathway name	Combined analysis statistics
Signaling molecules and interactions					
hsa05315	Neuroactive ligand-receptor interaction	*Χ*^2^ = 27.63, *p* <.001	hsa0460	Cytokine-cytokine receptor interaction	*Χ*^2^ = 17.75, *p* = .008
Nervous system					
hsa04726	Serotonergic synapse	*Χ*^2^ = 18.08, *p* = .008			
Cancer: overview					
hsa05200	Pathways in cancer	*Χ*^2^ = 23.24, *p* <.001	hsa05202	Transcriptional misregulation in cancer	*Χ*^2^ = 25.28, *p* = .001
Signal transduction					
hsa04151	PI3K-Akt signaling pathway	*Χ*^2^ = 17.91, *p* = .008			
hsa04010	MAPK signaling pathway	*Χ*^2^ = 17.71, *p* = .008			
Neurodegenerative disease					
hsa05022	Pathways of neurodegeneration — multiple diseases	*Χ*^2^ = 20.47, *p* = .004	hsa05020	Prion disease	*Χ*^2^ = 18.55, *p* = .006
hsa05010	Alzheimer disease	*Χ*^2^ = 23.74, *p* = .001	hsa05016	Huntington disease	*Χ*^2^ = 17.46, *p* = .008
Circulatory system					
hsa04270	Vascular smooth muscle contraction	*Χ*^2^ = 17.49, *p* = .008	hsa04260	Cardiac muscle contraction	*Χ*^2^ = 233.24, *p* = .001

Pathways are organized using the Kyoto Encyclopedia of Genes and Genomes database categories

*p* global perturbation *p*-value adjusted using the Benjamini-Hochberg procedure, *hsa* homo sapiens, *ID* identifier, *KEGG* Kyoto Encyclopedia of Genes and Genomes, *MAPK* mitogen-activated protein kinase, *PI3K-Akt* phosphoinositide-3-kinase/Akt

## Discussion

This study is the first to describe perturbations in signaling pathways associated with state anxiety in oncology patients receiving chemotherapy. This “Discussion” focuses on the eight common signaling pathways identified in this study and the network pharmacology studies [15–22], as well as the five additional perturbed pathways that were unique to the current study. The “Discussion” is organized using the six KEGG database categories listed above.

### Signaling molecules and interaction

The neuroactive ligand-receptor interaction pathway illustrates various ligand-receptor interactions that are critical components of cellular communication processes (e.g., proliferation, apoptosis) and cellular homeostasis [43]. This pathway includes a number of neurotransmitters associated with anxiety (e.g., 5-hydroxytryptamine [44], dopamine [45]) and that it was identified across the eight network pharmacology studies [15–22] suggests that it is an important mechanistic pathway for anxiety.

While not identified in the network pharmacology studies [15–22], support for the cytokine-cytokine receptor interaction pathway comes from studies of patients with colorectal cancer [46] and healthy volunteers [47], which found that higher levels of state anxiety were associated with higher levels of serum cytokines. In addition, findings from a preclinical study suggest that anxiety-like behaviors in mice are controlled through cytokine signaling from meningeal T cells as well as from peripherally derived cytokines [48]. As noted in one review [49], studies on the role of inflammation in anxiety are an area of emerging research.

### Nervous system

In terms of the serotonergic synapse pathway, 5-hydroxytryptamine is an important neurotransmitter that modulates a variety of physiologic processes, including emotions and behaviors [50]. The fact that this pathway was identified in the current study and across all eight of the network pharmacology studies [15–22] is not surprising given that selective serotonin reuptake inhibitors (SSRIs) are first-line treatment for anxiety disorders. However, the mechanisms by which SSRIs reduce anxiety are not fully understood [51]. Some mechanistic hypotheses include their effect(s) on fear learning circuits or blunting of emotional response(s) [51]. Of note, cancer treatment(s) may dysregulate the serotonergic synapse pathway. In a preclinical study that investigated the effects of combined radiotherapy and chemotherapy on emotional and cognitive function in mice at 5 and 15 weeks [52], long-term disruptions in hippocampal serotonergic signaling were identified. The authors hypothesized that the oxidative stress from chemotherapy produced marked reductions in serotonin 1AR receptor expression in several regions of the hippocampus that resulted in decreased serotonin levels and associated increases in anxiety-like behaviors observed in the mice.

### Cancer: overview

Pathways in cancer and transcriptional misregulation in cancer are two complex pathways identified in the current study that were associated with anxiety. While the complexity of both of these pathways limits the generation of specific mechanistic hypotheses, given that pathways in cancer were identified in three of the network pharmacology studies [18, 19, 22], additional research is warranted on associations between anxiety and various candidate genes in this pathway.

### Signal transduction

The PI3K/Akt and MAPK signaling pathways are involved in a variety of biological processes (e.g., protein synthesis, cellular proliferation, cell death [53, 54]). Each of these pathways was identified in two of the network pharmacology studies [17, 19]. In a review that evaluated anxiety-related microRNAs and their target transcripts, as well as described critical cellular pathways that underlie anxiety processing in the brain [53], nine anxiolytic and ten anxiogenic anxiety-related microRNAs were identified. Based on common hub genes, the PI3K/Akt and MAPK pathways were among the main signaling pathways affected by both anxiolytic and anxiogenic microRNAs. These findings suggest that a common feature across anxiety disorders may be dysregulation of these pathways. However, the authors noted that given the complexity of these pathways, more research is needed to identify how to effectively target signaling nodes within these pathways to reduce anxiety [53].

### Neurodegenerative disease

Pathways of neurodegeneration–multiple diseases are composed of a number of pathways. Therefore, specific hypotheses cannot be made about how this pathway may contribute to anxiety. However, anxiety is a common symptom reported by patients with neurodegenerative diseases [55–57]. Given the known neurodegenerative effects of chemotherapy [58, 59], it is plausible that shared mechanisms underlie anxiety in patients receiving chemotherapy and in patients with neurodegenerative diseases. This pathway was identified in one of the network pharmacology studies [22].

The finding of an association with the AD pathway is interesting given that anxiety is a risk factor for AD, a prodromal symptom of AD, and a common symptom associated with AD (i.e., pooled prevalence of 39%) [60]. This pathway was identified in one of the network pharmacology studies [16]. In terms of anxiety being a risk factor for AD, anxiety-related hypothalamic-pituitary-adrenal axis dysregulation and subsequent increases in glucocorticoids result in hippocampal atrophy and may increase the risk for dementia [61]. In addition, as noted in one review [60], a number of factors may contribute to the association between anxiety and AD, including neurobiological factors (e.g., increased salience network activity); psychosocial factors (e.g., unmet needs, awareness of cognitive, and functional impairments); associations between anxiety and cognitive impairment (e.g., increased severity of one symptom as a result of the presence of the other symptom); and environmental stressors (e.g., stimuli).

The association between anxiety and the Huntington disease pathway is interesting given that anxiety occurs in 13 to 71% of patients with this condition [62]. Of note, anxiety can occur up to ten years prior to the onset of the movement-related symptoms of this disease [63]. While the mechanisms that underlie this association are not fully understood, current treatment recommendations focus on the management of co-occurring symptoms (e.g., depression, sleep disturbance) that may contribute to the occurrence of anxiety [55].

The prion disease pathway represents a group of diseases known as prion diseases (e.g., Cruetzfeldt-Jacob disease, fatal insomnia, variably protease-sensitive prionopathy) [64]. While various symptoms are associated with prion diseases, changes in mood (e.g., anxiety) are common [65, 66]. However, little is known about the mechanisms that underlie anxiety in these conditions [67].

### Circulatory system

The association between anxiety and the vascular smooth muscle contraction pathway is interesting given that anxiety-regulating circuits within the amygdala and cerebral cortex play a role in mediating sympathetic nervous system responses that direct contraction of vascular smooth muscles [68]. This pathway was identified in one of the network pharmacology studies [18]. In the current study, the additional cardiac muscle contraction pathway was perturbed. This pathway represents the process by which the heart contracts. While not evaluated in patients with cancer, in a study of healthy college students, increases in state anxiety were associated with increases in heart rate fluctuations (i.e., both interbeat interval and its variation) [69].

### Limitations

Some limitations warrant consideration. While changes in state anxiety were assessed over two cycles of chemotherapy, blood was collected only at the enrollment assessment, and information on patients’ use of anxiolytics was not available. Longitudinal studies are needed that collect both phenotypic and molecular data to determine if pathway perturbations change over time. In addition, studies of a variety of biomarkers (e.g., epigenetic markers) are warranted to elucidate additional mechanisms for anxiety. Because this study is the first to evaluate for perturbed signaling pathways associated with anxiety in oncology patients, findings warrant confirmation within and among patients with different types of cancer.

### Conclusions and implications

This study evaluated for perturbed signaling pathways associated with low versus high levels of state anxiety in oncology patients. Given that the underlying mechanisms for anxiety may relate to how various anxiolytic drugs exert their effect(s) [70], our pathways were compared to a list of pathways identified in network pharmacology studies [15–22]. Taken together, these findings increase our knowledge of the molecular mechanisms that underlie anxiety in patients receiving chemotherapy. In addition, this study provides initial insights into how anxiety in patients with cancer may share common mechanisms with anxiety in patients with other clinical conditions. This knowledge is critical to the development of targeted interventions to decrease this devastating symptom.

### Supplementary information

ESM 1 (PDF 5 kb)

ESM 2 (DOCX 53 kb)

ESM 3 (DOCX 21 kb)

ESM 4 (DOCX 53 kb)

## Data Availability

Data will be provided to the publisher after they obtain a material transfer agreement from the University of California, San Francisco.
