# Supplementary material for: Anxiety in oncology outpatients is associated with perturbations in pathways identified in anxiety focused network pharmacology research
Source: Support Care Cancer. 2023 Nov 28;31(12):727. doi: 10.1007/s00520-023-08196-2 (PMC10682221; doi:10.1007/s00520-023-08196-2)
Supplement: Supplementary file 1 — (PDF 5 kb) [file 520_2023_8196_MOESM1_ESM.pdf]

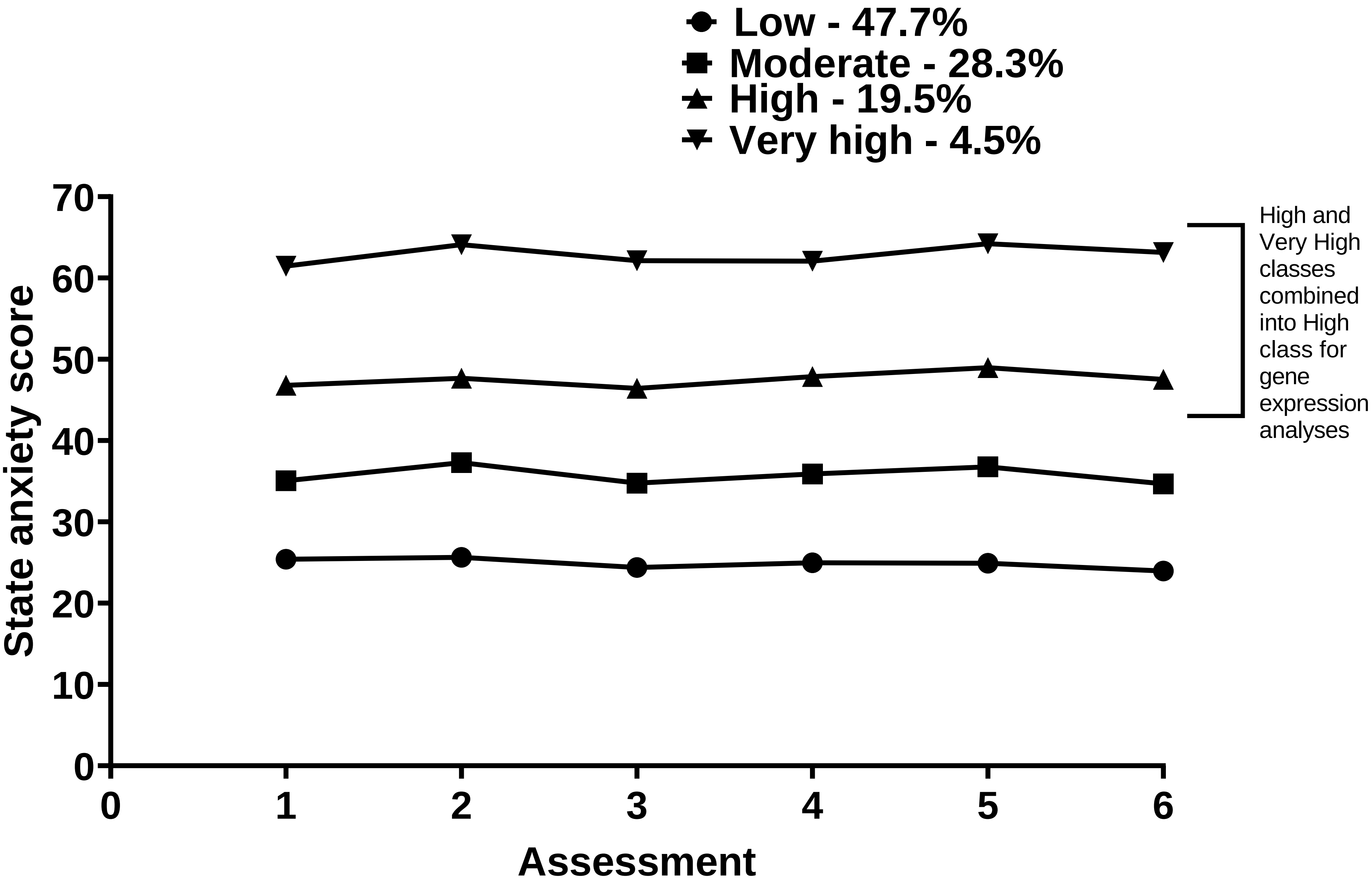

Supplemental Figure 1 - Latent class analysis of state anxiety scores across two cycles of chemotherapy (i.e., assessments 1 and 4 prior to the next dose of chemotherapy, assessments 2 and 5 approximately one week after the administration of chemotherapy, assessments 3 and 6 approximately two weeks after the administration of chemotherapy).
