# Supplementary material for: Anxiety in oncology outpatients is associated with perturbations in pathways identified in anxiety focused network pharmacology research
Source: Support Care Cancer. 2023 Nov 28;31(12):727. doi: 10.1007/s00520-023-08196-2 (PMC10682221; doi:10.1007/s00520-023-08196-2)
Supplement: Supplementary file 2 — (DOCX 53 kb) [file 520_2023_8196_MOESM2_ESM.docx]

717 patients provided a blood sample

Excluded patients in the Moderate class (n = 108) or with missing anxiety data (n = 2)

Excluded patients in the Moderate class (n = 99) or with missing anxiety data (n = 2)

Supplementary Figure 2: Flow diagram of the number of patients available for the gene expression analyses that evaluated for perturbations between the Low Anxiety and High Anxiety latent classes.

Abbreviations: GE = gene expression; RNA-seq = ribonucleic acid sequencing

Evaluable patients

Low anxiety = 157

High anxiety = 99

Evaluable patients

Low anxiety = 152

High anxiety = 92

GE analysis

Phenotypic analysis

Evaluable patients

n = 256

Evaluable patients

n = 244

Excluded after imputation n = 0

Microarray sample

n = 256

Excluded for poor quantification n = 3

Microarray sample

n = 259

1343 patients in the parent study

Microarray sample

n = 360

Quality control for phenotypic data

Patients with phenotypic data

Patients with the extreme phenotypes

Quality control for GE methodology

Excluded after imputation n = 0

RNA-seq sample

n = 244

Excluded for poor quantification n = 3

RNA-seq sample

n = 247

GE methodology

RNA-seq sample

n = 357
