# Supplementary material for: Anxiety in oncology outpatients is associated with perturbations in pathways identified in anxiety focused network pharmacology research
Source: Support Care Cancer. 2023 Nov 28;31(12):727. doi: 10.1007/s00520-023-08196-2 (PMC10682221; doi:10.1007/s00520-023-08196-2)
Supplement: Supplementary file 3 — (DOCX 21 kb) [file 520_2023_8196_MOESM3_ESM.docx]

Supplemental Table 1. Pathway Impact Analysis Results for the Low Anxiety (Low) Versus High Anxiety (High) Classes That Met the Global FDR Cutoff

| Pathway ID | KEGG pathway names | pPert RNA-seq | pPert  micora | Global X^2^ | Global FDR |
| --- | --- | --- | --- | --- | --- |
| hsa05310 | Asthma | <0.001 | <0.001 | 30.41 | <0.001 |
| hsa05311 | Phagosome | <0.001 | 0.001 | 29.02 | <0.001 |
| hsa05312 | Antigen processing and presentation | 0.001 | <0.001 | 29.02 | <0.001 |
| hsa05313 | Systemic lupus erythematosus | 0.001 | <0.001 | 29.02 | <0.001 |
| hsa05314 | Type I diabetes mellitus | 0.001 | <0.001 | 28.21 | <0.001 |
| hsa05315 | Neuroactive ligand-receptor interaction | <0.001 | 0.002 | 27.63 | <0.001 |
| hsa04672 | Intestinal immune network for IgA production | 0.002 | <0.001 | 27.63 | <0.001 |
| hsa05330 | Allograft rejection | 0.002 | <0.001 | 27.63 | <0.001 |
| hsa04144 | Endocytosis | 0.002 | <0.001 | 27.19 | <0.001 |
| hsa05332 | Graft-versus-host disease | 0.002 | <0.001 | 27.19 | <0.001 |
| hsa05150 | Staphylococcus aureus infection | <0.001 | 0.003 | 26.82 | <0.001 |
| hsa05323 | Rheumatoid arthritis | <0.001 | 0.005 | 25.61 | 0.001 |
| hsa04659 | Th17 cell differentiation | 0.006 | <0.001 | 25.44 | 0.001 |
| hsa05320 | Autoimmune thyroid disease | 0.001 | 0.003 | 25.44 | 0.001 |
| hsa05202 | Transcriptional misregulation in cancer | <0.001 | 0.006 | 25.28 | 0.001 |
| hsa05166 | Human T-cell leukemia virus 1 infection | 0.004 | 0.001 | 24.86 | 0.001 |
| hsa05010 | Alzheimer disease | 0.007 | 0.001 | 23.74 | 0.001 |
| hsa05412 | Arrhythmogenic right ventricular cardiomyopathy | <0.001 | 0.017 | 23.30 | 0.001 |
| hsa04260 | Cardiac muscle contraction | 0.001 | 0.006 | 23.24 | 0.001 |
| hsa05200 | Pathways in cancer | <0.001 | 0.018 | 23.24 | 0.001 |
| hsa05416 | Viral myocarditis | 0.002 | 0.004 | 23.03 | 0.001 |
| hsa05140 | Leishmaniasis | 0.003 | 0.004 | 22.12 | 0.002 |
| hsa05169 | Epstein-Barr virus infection | 0.004 | 0.005 | 21.45 | 0.002 |
| hsa01523 | Antifolate resistance | 0.028 | 0.001 | 20.93 | 0.003 |
| hsa05022 | Pathways of neurodegeneration - multiple diseases | 0.008 | 0.004 | 20.47 | 0.004 |
| hsa04210 | Apoptosis | 0.009 | 0.004 | 20.36 | 0.004 |
| hsa05171 | Coronavirus disease - COVID-19 | 0.048 | 0.001 | 19.89 | 0.004 |
| hsa04216 | Ferroptosis | 0.002 | 0.023 | 19.49 | 0.005 |
| hsa05410 | Hypertrophic cardiomyopathy | 0.001 | 0.062 | 19.38 | 0.005 |
| hsa04510 | Focal adhesion | 0.004 | 0.014 | 19.35 | 0.005 |
| hsa04650 | Natural killer cell mediated cytotoxicity | 0.072 | 0.001 | 19.07 | 0.005 |
| hsa05020 | Prion disease | 0.047 | 0.002 | 18.55 | 0.007 |
| hsa05168 | Herpes simplex virus 1 infection | <0.001 | 0.225 | 18.18 | 0.008 |
| hsa04726 | Serotonergic synapse | <0.001 | 0.238 | 18.08 | 0.008 |
| hsa05414 | Dilated cardiomyopathy | <0.001 | 0.255 | 17.94 | 0.008 |
| hsa04151 | PI3K-Akt signaling pathway | 0.003 | 0.043 | 17.91 | 0.008 |
| hsa04060 | Cytokine-cytokine receptor interaction | <0.001 | 0.280 | 17.75 | 0.008 |
| hsa04010 | MAPK signaling pathway | 0.011 | 0.013 | 17.71 | 0.008 |
| hsa05145 | Toxoplasmosis | 0.005 | 0.028 | 17.52 | 0.008 |
| hsa04270 | Vascular smooth muscle contraction | 0.011 | 0.014 | 17.49 | 0.008 |
| hsa05016 | Huntington disease | 0.323 | <0.001 | 17.46 | 0.008 |

Abbreviations: FDR = false discovery rate; hsa = homo sapiens; ID = identifier; KEGG = Kyoto Encyclopedia of Genes and Genomes; microa = microarray sample; pPert = probability of pathway perturbations; RNA-seq = ribonucleic acid sequencing sample

Note: Global FDR adjusted using the Benjamini-Hochberg procedure
