# Supplementary material for: Anxiety in oncology outpatients is associated with perturbations in pathways identified in anxiety focused network pharmacology research
Source: Support Care Cancer. 2023 Nov 28;31(12):727. doi: 10.1007/s00520-023-08196-2 (PMC10682221; doi:10.1007/s00520-023-08196-2)
Supplement: Supplementary file 4 — (DOCX 53 kb) [file 520_2023_8196_MOESM4_ESM.docx]

Supplemental Table 2. Pathway Impact Analysis Results for All of the Pathways Identified for the Low Anxiety (Low) Versus High Anxiety (High) Classes

| Pathway ID | KEGG pathway names | pPert RNA-seq | pPert  micora | Global X^2^ | Global FDR |
| --- | --- | --- | --- | --- | --- |
| hsa05310 | Asthma | <0.001 | <0.001 | 30.41 | <0.001 |
| hsa05311 | Phagosome | <0.001 | 0.001 | 29.02 | <0.001 |
| hsa05312 | Antigen processing and presentation | 0.001 | <0.001 | 29.02 | <0.001 |
| hsa05313 | Systemic lupus erythematosus | 0.001 | <0.001 | 29.02 | <0.001 |
| hsa05314 | Type I diabetes mellitus | 0.001 | <0.001 | 28.21 | <0.001 |
| hsa05315 | Neuroactive ligand-receptor interaction | <0.001 | 0.002 | 27.63 | <0.001 |
| hsa04672 | Intestinal immune network for IgA production | 0.002 | <0.001 | 27.63 | <0.001 |
| hsa05330 | Allograft rejection | 0.002 | <0.001 | 27.63 | <0.001 |
| hsa04144 | Endocytosis | 0.002 | <0.001 | 27.19 | <0.001 |
| hsa05332 | Graft-versus-host disease | 0.002 | <0.001 | 27.19 | <0.001 |
| hsa05150 | Staphylococcus aureus infection | <0.001 | 0.003 | 26.82 | <0.001 |
| hsa05323 | Rheumatoid arthritis | <0.001 | 0.005 | 25.61 | 0.001 |
| hsa04659 | Th17 cell differentiation | 0.006 | <0.001 | 25.44 | 0.001 |
| hsa05320 | Autoimmune thyroid disease | 0.001 | 0.003 | 25.44 | 0.001 |
| hsa05202 | Transcriptional misregulation in cancer | <0.001 | 0.006 | 25.28 | 0.001 |
| hsa05166 | Human T-cell leukemia virus 1 infection | 0.004 | 0.001 | 24.86 | 0.001 |
| hsa05010 | Alzheimer disease | 0.007 | 0.001 | 23.74 | 0.001 |
| hsa05412 | Arrhythmogenic right ventricular cardiomyopathy | <0.001 | 0.017 | 23.30 | 0.001 |
| hsa04260 | Cardiac muscle contraction | 0.001 | 0.006 | 23.24 | 0.001 |
| hsa05200 | Pathways in cancer | <0.001 | 0.018 | 23.24 | 0.001 |
| hsa05416 | Viral myocarditis | 0.002 | 0.004 | 23.03 | 0.001 |
| hsa05140 | Leishmaniasis | 0.003 | 0.004 | 22.12 | 0.002 |
| hsa05169 | Epstein-Barr virus infection | 0.004 | 0.005 | 21.45 | 0.002 |
| hsa01523 | Antifolate resistance | 0.028 | 0.001 | 20.93 | 0.003 |
| hsa05022 | Pathways of neurodegeneration - multiple diseases | 0.008 | 0.004 | 20.47 | 0.004 |
| hsa04210 | Apoptosis | 0.009 | 0.004 | 20.36 | 0.004 |
| hsa05171 | Coronavirus disease - COVID-19 | 0.048 | 0.001 | 19.89 | 0.004 |
| hsa04216 | Ferroptosis | 0.002 | 0.023 | 19.49 | 0.005 |
| hsa05410 | Hypertrophic cardiomyopathy | 0.001 | 0.062 | 19.38 | 0.005 |
| hsa04510 | Focal adhesion | 0.004 | 0.014 | 19.35 | 0.005 |
| hsa04650 | Natural killer cell mediated cytotoxicity | 0.072 | 0.001 | 19.07 | 0.005 |
| hsa05020 | Prion disease | 0.047 | 0.002 | 18.55 | 0.007 |
| hsa05168 | Herpes simplex virus 1 infection | <0.001 | 0.225 | 18.18 | 0.008 |
| hsa04726 | Serotonergic synapse | <0.001 | 0.238 | 18.08 | 0.008 |
| hsa05414 | Dilated cardiomyopathy | <0.001 | 0.255 | 17.94 | 0.008 |
| hsa04151 | PI3K-Akt signaling pathway | 0.003 | 0.043 | 17.91 | 0.008 |
| hsa04060 | Cytokine-cytokine receptor interaction | <0.001 | 0.280 | 17.75 | 0.008 |
| hsa04010 | MAPK signaling pathway | 0.011 | 0.013 | 17.71 | 0.008 |
| hsa05145 | Toxoplasmosis | 0.005 | 0.028 | 17.52 | 0.008 |
| hsa04270 | Vascular smooth muscle contraction | 0.011 | 0.014 | 17.49 | 0.008 |
| hsa05016 | Huntington disease | 0.323 | <0.001 | 17.46 | 0.008 |
| hsa05014 | Amyotrophic lateral sclerosis | 0.417 | <0.001 | 16.95 | 0.010 |
| hsa05165 | Human papillomavirus infection | 0.001 | 0.211 | 16.93 | 0.010 |
| hsa04217 | Necroptosis | 0.011 | 0.020 | 16.85 | 0.010 |
| hsa04064 | NF-kappa B signaling pathway | 0.069 | 0.004 | 16.39 | 0.012 |
| hsa05215 | Prostate cancer | 0.005 | 0.064 | 16.08 | 0.014 |
| hsa04621 | NOD-like receptor signaling pathway | 0.003 | 0.095 | 16.02 | 0.014 |
| hsa04723 | Retrograde endocannabinoid signaling | 0.017 | 0.022 | 15.74 | 0.015 |
| hsa05220 | Chronic myeloid leukemia | 0.772 | <0.001 | 15.72 | 0.015 |
| hsa05164 | Influenza A | 0.006 | 0.061 | 15.67 | 0.015 |
| hsa04630 | JAK-STAT signaling pathway | 0.019 | 0.021 | 15.66 | 0.015 |
| hsa04141 | Protein processing in endoplasmic reticulum | 0.034 | 0.012 | 15.58 | 0.015 |
| hsa05162 | Measles | 0.019 | 0.022 | 15.47 | 0.016 |
| hsa04660 | T cell receptor signaling pathway | 0.962 | <0.001 | 15.28 | 0.017 |
| hsa04350 | TGF-beta signaling pathway | <0.001 | 0.972 | 15.26 | 0.017 |
| hsa05144 | Malaria | 0.005 | 0.090 | 15.21 | 0.017 |
| hsa05152 | Tuberculosis | 0.005 | 0.102 | 15.16 | 0.017 |
| hsa04979 | Cholesterol metabolism | 0.017 | 0.030 | 15.13 | 0.017 |
| hsa04110 | Cell cycle | 0.005 | 0.105 | 14.91 | 0.018 |
| hsa04014 | Ras signaling pathway | 0.058 | 0.011 | 14.72 | 0.020 |
| hsa04910 | Insulin signaling pathway | 0.373 | 0.002 | 14.40 | 0.022 |
| hsa05142 | Chagas disease | 0.533 | 0.001 | 14.27 | 0.023 |
| hsa04380 | Osteoclast differentiation | 0.041 | 0.02 | 14.14 | 0.024 |
| hsa05170 | Human immunodeficiency virus 1 infection | 0.851 | 0.001 | 14.14 | 0.024 |
| hsa04727 | GABAergic synapse | 0.002 | 0.438 | 14.08 | 0.024 |
| hsa05207 | Chemical carcinogenesis - receptor activation | 0.006 | 0.141 | 13.99 | 0.025 |
| hsa04024 | cAMP signaling pathway | 0.001 | 0.943 | 13.93 | 0.025 |
| hsa05203 | Viral carcinogenesis | 0.138 | 0.007 | 13.89 | 0.025 |
| hsa05417 | Lipid and atherosclerosis | 0.132 | 0.007 | 13.83 | 0.025 |
| hsa04068 | FoxO signaling pathway | 0.038 | 0.027 | 13.77 | 0.026 |
| hsa05206 | MicroRNAs in cancer | 0.019 | 0.053 | 13.73 | 0.026 |
| hsa04610 | Complement and coagulation cascades | 0.041 | 0.026 | 13.65 | 0.026 |
| hsa05017 | Spinocerebellar ataxia | 0.390 | 0.003 | 13.50 | 0.027 |
| hsa05235 | PD-L1 expression and PD-1 checkpoint pathway in cancer | 0.843 | 0.001 | 13.35 | 0.029 |
| hsa05321 | Inflammatory bowel disease | 0.029 | 0.044 | 13.30 | 0.029 |
| hsa04664 | Fc epsilon RI signaling pathway | 0.068 | 0.022 | 13.00 | 0.033 |
| hsa04390 | Hippo signaling pathway | 0.004 | 0.389 | 12.93 | 0.033 |
| hsa05135 | Yersinia infection | 0.807 | 0.002 | 12.86 | 0.034 |
| hsa04512 | ECM-receptor interaction | 0.004 | 0.412 | 12.82 | 0.034 |
| hsa05012 | Parkinson disease | 0.859 | 0.002 | 12.73 | 0.035 |
| hsa04666 | Fc gamma R-mediated phagocytosis | 0.190 | 0.009 | 12.63 | 0.036 |
| hsa04020 | Calcium signaling pathway | 0.002 | 0.929 | 12.58 | 0.036 |
| hsa04146 | Peroxisome | 0.038 | 0.050 | 12.49 | 0.037 |
| hsa04360 | Axon guidance | 0.003 | 0.556 | 12.49 | 0.037 |
| hsa04934 | Cushing syndrome | 0.003 | 0.639 | 12.52 | 0.037 |
| hsa04933 | AGE-RAGE signaling pathway in diabetic complications | 0.033 | 0.063 | 12.34 | 0.038 |
| hsa04972 | Pancreatic secretion | 0.032 | 0.065 | 12.35 | 0.038 |
| hsa05131 | Shigellosis | 0.847 | 0.002 | 12.32 | 0.038 |
| hsa04137 | Mitophagy - animal | 0.119 | 0.018 | 12.29 | 0.038 |
| hsa04658 | Th1 and Th2 cell differentiation | 0.331 | 0.007 | 12.00 | 0.043 |
| hsa04912 | GnRH signaling pathway | 0.034 | 0.075 | 11.95 | 0.043 |
| hsa04810 | Regulation of actin cytoskeleton | 0.005 | 0.517 | 11.92 | 0.043 |
| hsa04929 | GnRH secretion | 0.004 | 0.599 | 11.83 | 0.044 |
| hsa05226 | Gastric cancer | 0.004 | 0.653 | 11.66 | 0.047 |
| hsa04072 | Phospholipase D signaling pathway | 0.034 | 0.087 | 11.61 | 0.048 |
| hsa04130 | SNARE interactions in vesicular transport | 0.021 | 0.147 | 11.56 | 0.048 |
| hsa04215 | Apoptosis - multiple species | 0.006 | 0.569 | 11.36 | 0.051 |
| hsa04724 | Glutamatergic synapse | 0.005 | 0.635 | 11.32 | 0.051 |
| hsa04927 | Cortisol synthesis and secretion | 0.004 | 0.785 | 11.29 | 0.051 |
| hsa05143 | African trypanosomiasis | 0.005 | 0.704 | 11.30 | 0.051 |
| hsa05146 | Amoebiasis | 0.026 | 0.130 | 11.37 | 0.051 |
| hsa01521 | EGFR tyrosine kinase inhibitor resistance | 0.004 | 0.921 | 11.21 | 0.051 |
| hsa03018 | RNA degradation | 0.925 | 0.004 | 11.20 | 0.051 |
| hsa04012 | ErbB signaling pathway | 0.008 | 0.426 | 11.24 | 0.051 |
| hsa04150 | mTOR signaling pathway | 0.006 | 0.562 | 11.23 | 0.051 |
| hsa04916 | Melanogenesis | 0.007 | 0.500 | 11.17 | 0.051 |
| hsa05205 | Proteoglycans in cancer | 0.005 | 0.76 | 10.96 | 0.055 |
| hsa05217 | Basal cell carcinoma | 0.005 | 0.755 | 10.97 | 0.055 |
| hsa04742 | Taste transduction | 0.006 | 0.671 | 10.87 | 0.057 |
| hsa05221 | Acute myeloid leukemia | 0.193 | 0.024 | 10.71 | 0.060 |
| hsa04935 | Growth hormone synthesis, secretion and action | 0.044 | 0.117 | 10.53 | 0.064 |
| hsa05225 | Hepatocellular carcinoma | 0.006 | 0.801 | 10.52 | 0.064 |
| hsa05415 | Diabetic cardiomyopathy | 0.247 | 0.023 | 10.30 | 0.070 |
| hsa04930 | Type II diabetes mellitus | 0.008 | 0.697 | 10.26 | 0.070 |
| hsa05231 | Choline metabolism in cancer | 0.016 | 0.360 | 10.25 | 0.070 |
| hsa04213 | Longevity regulating pathway - multiple species | 0.585 | 0.011 | 10.09 | 0.074 |
| hsa04611 | Platelet activation | 0.073 | 0.088 | 10.08 | 0.074 |
| hsa04530 | Tight junction | 0.008 | 0.792 | 10.00 | 0.076 |
| hsa05223 | Non-small cell lung cancer | 0.063 | 0.109 | 9.96 | 0.076 |
| hsa05132 | Salmonella infection | 0.877 | 0.008 | 9.92 | 0.077 |
| hsa04022 | cGMP-PKG signaling pathway | 0.023 | 0.314 | 9.82 | 0.08 |
| hsa05134 | Legionellosis | 0.630 | 0.012 | 9.77 | 0.081 |
| hsa05224 | Breast cancer | 0.009 | 0.854 | 9.74 | 0.081 |
| hsa05032 | Morphine addiction | 0.019 | 0.403 | 9.69 | 0.082 |
| hsa04921 | Oxytocin signaling pathway | 0.012 | 0.688 | 9.59 | 0.085 |
| hsa05222 | Small cell lung cancer | 0.012 | 0.688 | 9.51 | 0.087 |
| hsa04919 | Thyroid hormone signaling pathway | 0.021 | 0.430 | 9.42 | 0.090 |
| hsa04261 | Adrenergic signaling in cardiomyocytes | 0.014 | 0.659 | 9.30 | 0.093 |
| hsa04973 | Carbohydrate digestion and absorption | 0.012 | 0.814 | 9.26 | 0.094 |
| hsa04978 | Mineral absorption | 0.121 | 0.081 | 9.25 | 0.094 |
| hsa04913 | Ovarian steroidogenesis | 0.023 | 0.442 | 9.18 | 0.096 |
| hsa04015 | Rap1 signaling pathway | 0.034 | 0.303 | 9.15 | 0.096 |
| hsa05212 | Pancreatic cancer | 0.835 | 0.012 | 9.13 | 0.096 |
| hsa04330 | Notch signaling pathway | 0.305 | 0.035 | 9.05 | 0.099 |
| hsa04725 | Cholinergic synapse | 0.056 | 0.202 | 8.97 | 0.101 |
| hsa04714 | Thermogenesis | 0.506 | 0.023 | 8.86 | 0.103 |
| hsa04915 | Estrogen signaling pathway | 0.012 | 0.945 | 8.88 | 0.103 |
| hsa04924 | Renin secretion | 0.013 | 0.880 | 8.87 | 0.103 |
| hsa05100 | Bacterial invasion of epithelial cells | 0.342 | 0.036 | 8.77 | 0.107 |
| hsa04932 | Non-alcoholic fatty liver disease | 0.790 | 0.016 | 8.74 | 0.107 |
| hsa04925 | Aldosterone synthesis and secretion | 0.014 | 0.885 | 8.71 | 0.108 |
| hsa04540 | Gap junction | 0.021 | 0.644 | 8.61 | 0.112 |
| hsa04062 | Chemokine signaling pathway | 0.027 | 0.506 | 8.59 | 0.112 |
| hsa04911 | Insulin secretion | 0.017 | 0.810 | 8.51 | 0.114 |
| hsa05218 | Melanoma | 0.019 | 0.743 | 8.52 | 0.114 |
| hsa01524 | Platinum drug resistance | 0.015 | 0.975 | 8.45 | 0.116 |
| hsa05130 | Pathogenic Escherichia coli infection | 0.666 | 0.024 | 8.23 | 0.125 |
| hsa04721 | Synaptic vesicle cycle | 0.043 | 0.402 | 8.10 | 0.132 |
| hsa04976 | Bile secretion | 0.030 | 0.580 | 8.07 | 0.132 |
| hsa04115 | p53 signaling pathway | 0.039 | 0.491 | 7.91 | 0.140 |
| hsa04310 | Wnt signaling pathway | 0.045 | 0.455 | 7.76 | 0.147 |
| hsa05161 | Hepatitis B | 0.060 | 0.339 | 7.77 | 0.147 |
| hsa04920 | Adipocytokine signaling pathway | 0.109 | 0.194 | 7.70 | 0.149 |
| hsa04950 | Maturity onset diabetes of the young | 0.212 | 0.101 | 7.68 | 0.150 |
| hsa05120 | Epithelial cell signaling in Helicobacter pylori infection | 0.398 | 0.058 | 7.54 | 0.157 |
| hsa04928 | Parathyroid hormone synthesis, secretion and action | 0.047 | 0.511 | 7.46 | 0.161 |
| hsa04728 | Dopaminergic synapse | 0.053 | 0.547 | 7.06 | 0.187 |
| hsa05210 | Colorectal cancer | 0.068 | 0.454 | 6.94 | 0.194 |
| hsa04670 | Leukocyte transendothelial migration | 0.040 | 0.811 | 6.86 | 0.200 |
| hsa04961 | Endocrine and other factor-regulated calcium reabsorption | 0.042 | 0.893 | 6.57 | 0.222 |
| hsa04370 | VEGF signaling pathway | 0.170 | 0.244 | 6.36 | 0.239 |
| hsa04713 | Circadian entrainment | 0.094 | 0.450 | 6.32 | 0.240 |
| hsa05031 | Amphetamine addiction | 0.049 | 0.872 | 6.31 | 0.240 |
| hsa05133 | Pertussis | 0.095 | 0.449 | 6.30 | 0.240 |
| hsa05034 | Alcoholism | 0.437 | 0.107 | 6.12 | 0.255 |
| hsa04970 | Salivary secretion | 0.077 | 0.733 | 5.75 | 0.29 |
| hsa05110 | Vibrio cholerae infection | 0.082 | 0.687 | 5.74 | 0.29 |
| hsa04623 | Cytosolic DNA-sensing pathway | 0.544 | 0.123 | 5.40 | 0.327 |
| hsa05167 | Kaposi sarcoma-associated herpesvirus infection | 0.605 | 0.128 | 5.11 | 0.361 |
| hsa05418 | Fluid shear stress and atherosclerosis | 0.255 | 0.332 | 4.94 | 0.382 |
| hsa04136 | Autophagy - other | 0.726 | 0.120 | 4.88 | 0.387 |
| hsa04066 | HIF-1 signaling pathway | 0.651 | 0.142 | 4.76 | 0.400 |
| hsa04730 | Long-term depression | 0.257 | 0.358 | 4.77 | 0.400 |
| hsa04071 | Sphingolipid signaling pathway | 0.761 | 0.135 | 4.55 | 0.427 |
| hsa04140 | Autophagy - animal | 0.975 | 0.112 | 4.42 | 0.445 |
| hsa04061 | Viral protein interaction with cytokine and cytokine receptor | 0.401 | 0.280 | 4.37 | 0.450 |
| hsa04613 | Neutrophil extracellular trap formation | 0.117 | 0.974 | 4.34 | 0.452 |
| hsa04662 | B cell receptor signaling pathway | 0.251 | 0.492 | 4.18 | 0.474 |
| hsa03440 | Homologous recombination | 0.159 | 0.794 | 4.14 | 0.478 |
| hsa04923 | Regulation of lipolysis in adipocytes | 0.602 | 0.218 | 4.06 | 0.488 |
| hsa04218 | Cellular senescence | 0.200 | 0.692 | 3.96 | 0.502 |
| hsa04625 | C-type lectin receptor signaling pathway | 0.223 | 0.624 | 3.95 | 0.502 |
| hsa04114 | Oocyte meiosis | 0.264 | 0.531 | 3.93 | 0.502 |
| hsa03320 | PPAR signaling pathway | 0.328 | 0.441 | 3.87 | 0.507 |
| hsa04620 | Toll-like receptor signaling pathway | 0.197 | 0.731 | 3.87 | 0.507 |
| hsa05219 | Bladder cancer | 0.961 | 0.159 | 3.76 | 0.522 |
| hsa04152 | AMPK signaling pathway | 0.314 | 0.497 | 3.71 | 0.527 |
| hsa04122 | Sulfur relay system | 0.309 | 0.512 | 3.69 | 0.529 |
| hsa04922 | Glucagon signaling pathway | 0.213 | 0.754 | 3.66 | 0.532 |
| hsa04971 | Gastric acid secretion | 0.276 | 0.609 | 3.57 | 0.544 |
| hsa04931 | Insulin resistance | 0.993 | 0.173 | 3.52 | 0.547 |
| hsa04962 | Vasopressin-regulated water reabsorption | 0.699 | 0.246 | 3.52 | 0.547 |
| hsa05211 | Renal cell carcinoma | 0.568 | 0.320 | 3.41 | 0.564 |
| hsa04926 | Relaxin signaling pathway | 0.289 | 0.656 | 3.32 | 0.575 |
| hsa04918 | Thyroid hormone synthesis | 0.298 | 0.730 | 3.05 | 0.623 |
| hsa03015 | mRNA surveillance pathway | 0.985 | 0.224 | 3.02 | 0.624 |
| hsa04340 | Hedgehog signaling pathway | 0.953 | 0.243 | 2.92 | 0.64 |
| hsa01522 | Endocrine resistance | 0.419 | 0.620 | 2.70 | 0.68 |
| hsa03460 | Fanconi anemia pathway | 0.612 | 0.431 | 2.67 | 0.683 |
| hsa05030 | Cocaine addiction | 0.726 | 0.401 | 2.47 | 0.718 |
| hsa04211 | Longevity regulating pathway | 0.468 | 0.721 | 2.17 | 0.774 |
| hsa04657 | IL-17 signaling pathway | 0.360 | 0.968 | 2.11 | 0.783 |
| hsa05213 | Endometrial cancer | 0.769 | 0.466 | 2.05 | 0.791 |
| hsa04914 | Progesterone-mediated oocyte maturation | 0.750 | 0.509 | 1.93 | 0.811 |
| hsa04371 | Apelin signaling pathway | 0.860 | 0.507 | 1.66 | 0.847 |
| hsa04710 | Circadian rhythm | 0.513 | 0.846 | 1.67 | 0.847 |
| hsa05163 | Human cytomegalovirus infection | 0.940 | 0.460 | 1.68 | 0.847 |
| hsa05230 | Central carbon metabolism in cancer | 0.851 | 0.505 | 1.69 | 0.847 |
| hsa05216 | Thyroid cancer | 0.943 | 0.490 | 1.55 | 0.866 |
| hsa04740 | Olfactory transduction | 0.710 | 0.696 | 1.41 | 0.886 |
| hsa04622 | RIG-I-like receptor signaling pathway | 0.644 | 0.817 | 1.29 | 0.905 |
| hsa05214 | Glioma | 0.609 | 0.892 | 1.22 | 0.912 |
| hsa04392 | Hippo signaling pathway - multiple species | 0.907 | 0.622 | 1.14 | 0.921 |
| hsa04722 | Neurotrophin signaling pathway | 0.696 | 0.849 | 1.05 | 0.931 |
| hsa04917 | Prolactin signaling pathway | 0.809 | 0.745 | 1.02 | 0.933 |
| hsa04614 | Renin-angiotensin system | 0.734 | 0.851 | 0.94 | 0.937 |
| hsa04720 | Long-term potentiation | 0.714 | 0.879 | 0.93 | 0.937 |
| hsa04960 | Aldosterone-regulated sodium reabsorption | 0.723 | 0.942 | 0.77 | 0.951 |
| hsa05160 | Hepatitis C | 0.768 | 0.875 | 0.80 | 0.951 |
| hsa04668 | TNF signaling pathway | 0.791 | 0.938 | 0.60 | 0.968 |
| hsa04744 | Phototransduction | 0.987 | 0.879 | 0.29 | 0.991 |

Abbreviations: FDR = false discovery rate; hsa = homo sapiens; ID = identifier; KEGG = Kyoto Encyclopedia of Genes and Genomes; microa = microarray sample; pPert = probability of pathway perturbations; RNA-seq = ribonucleic acid sequencing sample

Note: Global FDR adjusted using the Benjamini-Hochberg procedure
